# Assessment of the therapeutic efficacy of a paediatric formulation of artemether-lumefantrine (Coartesiane^®^) for the treatment of uncomplicated *Plasmodium falciparum *in children in Zambia

**DOI:** 10.1186/1475-2875-5-75

**Published:** 2006-08-28

**Authors:** Pascalina Chanda, Moonga Hawela, Mabvuto Kango, Naawa Sipilanyambe

**Affiliations:** 1 National Malaria Control Centre, P.O. Box 32509, Lusaka, Zambia

## Abstract

**Background:**

Sentinel site surveillance of antimalarials by in-vivo therapeutic efficacy studies in Zambia is one of the key activities ear-marked for monitoring and evaluation. The studies are conducted annually in order to provide timely and reliable information on the status of the recommended regimens for malaria case management. The findings of the therapeutic efficacy of an artemisinin-based combination therapy of pediatric artemether-lumefantrine (Coartesiane^®^) are reported.

**Method:**

The design is a simple, one-arm, prospective evaluation of the clinical and parasitological response to directly observed treatment for uncomplicated malaria. The study was conducted in sentinel sites using the WHO standardized protocol for the assessment of therapeutic efficacy of antimalarial drugs (WHO 2000) in children under five years of age, weighing less than 10 Kg. The study was conducted at two clinics, one in Chongwe (Lusaka Province) and Chipata (Eastern Province). The 28-day follow-up period was used coupled with PCR genotyping for MSP1 and MSP2 in order to differentiate recrudescence from re-infections for parasites that appeared after Day 14.

**Results:**

91/111 children enrolled in the study, were successfully followed up. Artemether-lumefantrine (Coartesiane^®^) was found to produce significant gametocyte reduction. The Adequate Clinical and Parasitological Response (ACPR) was found to be 100% (95% CI 96.0;100).

**Conclusion:**

Coartesiane^® ^was effective in treating uncomplicated malaria in Zambian children weighing less than 10 kg, an age group normally excluded from taking the tablet formulation of artemether-lumefantrine (Coartem^®^).

## Background

The malaria situation analysis in Zambia shows that malaria is the leading cause of mortality and morbidity, and the disease burden is worse in the biologically vulnerable children, under five years of age, and in pregnant women [1]. The development of drug resistance in Zambia has had a major impact on the success of malaria control. The country has experienced major treatment failures to chloroquine and sulphadoxine/pyrimethamine (SP), with recorded treatment failures for SP as high as 32.6% in certain parts of the country by early 2003 [2]. After the documentation of chloroquine resistance in 1983, the outpatient department (OPD) cases increased from 167 cases per thousand population to 428 cases per thousand population in 2003 [3]. The public health impact of drug resistance led to an increase in the disease prevalence rates, under five mortality rates and a corresponding increase in case fatality rates. This was evidenced by an upward surge in all epidemiological indicators of the malaria disease.

The assessment and monitoring of antimalarial therapeutic efficacy is key to the provision of sound evidence for policy decisions making regarding which antimalarials can be adopted for malaria case management. The National Malaria Control Centre has over time developed a consistent system for sentinel site surveillance of the therapeutic efficacy of antimalarial drugs. These studies have played a major role in guiding the country's decision to move from monotherapies to Artemisinin-Based Combination Therapies (ACTs).

The use of combination therapy is expected to have a positive impact on malaria transmission by lowering the rates of gametocytaemia after treatment. Artemisinin derivatives are advocated for use in antimalarial combination therapy because they quickly reduce the level of parasitaemia and hence the parasite pool from which resistant *P. falciparum *strains may arise [4].

But, as the new drug policy outlines, SP is the current recommended drug for intermittent presumptive treatment for malaria in pregnancy and for children weighing less than 10 Kg. These two groups were excluded from using the tablet formulation of artemether-lumefantrine (Coartem^®^), because there was insufficient safety and efficacy data at the time of the policy change.

The therapeutic efficacy of Coartesiane^® ^in treating uncomplicated *Plasmodium falciparum *malaria in children was assessed. The findings will be useful in providing evidence on the possible options for children who currently are being exposed to the ineffective SP.

## Methodology

### Study design

The study was an open label one arm prospective evaluation of the therapeutic efficacy of antimalarials in design. It was conducted between March to May 2005 in two sentinel sites, Chongwe and Chipata.

### Study population and sample size

The patients included were children under five years of age, weighing less than 10 Kg (the minimum weight admissible for Coartem^®^). Other criteria used for inclusion were: absence of severe malnutrition, mono-infection with *P. falciparum *, with a parasitaemia in the range of 2,000 to 200,000 asexual parasites per micro litre of blood, absence of severe malaria or danger signs, axillary temperature ≥ 37.5°C at visit, absence of other febrile conditions, ability to come for follow-up visit and informed consent from parent or guardian.

The classical statistical methods for determining sample size based on an expected proportion of treatment failures was used, with a 95% confidence level and a precision of 10%. The failure rates were expected to be lower than 15%, so a minimum of 50 patients had to be enrolled at each site.

### Parasitological examination

Two thick films were prepared before treatment on Day 0, and on Days 1, 2, 3, 7, 14, 21 and 28 including any other day that the patient was brought to the clinic before the next scheduled visit. One thick film was used for rapid staining (10–15 minutes in 10% Giemsa stain) while the second film was for slow staining (30–45 minutes in 3% Giemsa stain). The quick stain was used for initial screening for the presence of parasites, while the second blood smear was used to calculate parasite density. Parasitaemia (per microlitre) = number of parasites × 8,000/number of leucocytes counted.

Two laboratory technicians were engaged in the study and both read through all the slides independently to assure quality. All the slides for the patients on the study were collected and used in quality control by an independent laboratory.

### Treatment and follow-up

The study followed the standard World Health Organization (WHO) Protocol for the *Assessment of Therapeutic Efficacy of Antimalarial Drugs for Uncomplicated Plasmodium falciparum malaria *adapted to the Zambian situation to include studies with artemisinin-based combination therapy. The 28-day follow-up period was used.

On enrolment day, Day 0, a brief history of each child was obtained, the patient was weighed and the axillary temperature was recorded. The patient was then sent for laboratory examination to confirm malaria diagnosis and also to identify if parasitaemia was adequate for enrolment. Upon fulfillment of all inclusion criteria, consent was obtained from the caretaker and the child was enrolled on the study.

Coartesiane^® ^is an oral suspension of artemether (180 mg/60 ml) and lumefantrine (1080 mg/60 ml) indicated for the treatment of malaria in children. It is presented in a yellow powder which can be diluted with water to form a 60 ml suspension. The dosing schedule is based on body weight and it is to be taken once a day for three days. The drug is manufactured by Dafra Pharma nv/sa. The drug is registered in Zambia for use in children for the treatment of uncomplicated malaria and is being used in the private sector.

All medication was given under the supervision of the study team. Patients were observed for a few minutes after administering the study drug to ensure that they did not vomit. Any child who vomited had the dose re-administered but if vomiting persisted, the child was dropped from the study and referred for further management. A photograph of the child was taken as an identification tool on follow-up days and also as an incentive upon completion of the study.

Follow-ups were scheduled for Days 2, 3, 7, 14, 21 and 28 plus any other day that the child was taken to the clinic on unscheduled day. On each of these days clinical and parasitological assessments were performed. The community health worker ensured the directions to the home of the patient were well written for ease of tracking of patients.

Filter paper samples of blood were taken on Day 0 and any other day when the parasites first reappeared in the blood of the patient. Polymerase Chain Reaction (PCR) genotyping for MSP1 and MSP2 was done to differentiate recrudescence from re-infections. DNA was extracted from the filter papers and analysed using standard methods as described elsewhere [5,6]. A recrudescent infection was defined if the allelic bands of the subsequent infection were similar to the Day 0 pattern. However, a reinfection was defined if the allelic bands for the subsequent infection were different from the Day 0 bands.

Children were withdrawn from the study if the patient developed any signs of severe or complicated malaria or any of the general danger signs during the follow-up period; he/she was given the first dose of parenteral quinine and taken urgently to the appropriate health facility. For ethical reasons, all children with parasitaemia on Day 28, irrespective of symptoms were treated with the alternative antimalarial drug (oral quinine) at the end of the follow-up period.

### Incentives

Incentives were given to the caretakers as a way to motivate them not to miss a scheduled visit to the study site. A 1 Kg packet of sugar was given to each patient on Day 3, a bar of laundry soap on Day 7 and a packet of salt on Day 14 and a photograph of the child on Day 28. Any child, who dropped from the study before Day 28, received the photo on the last day of being on the study. Sweets were given to the children as an incentive for the number of finger pricks they get on the study.

### Data collection

The data was recorded depending on the day of the study. The screening Log sheet was kept in the laboratory and this had serial numbers and information of all the patients screened for the study irrespective of their parasitaemia and clinical condition. The Case enrolment form was used for the patient's case history and it also contained clinical and parasitological data for Day 0. The case record form was used to record the patients' study number, directions to home, study drug, and all the clinical and parasitological data for Day 0 to Day 28, and the final classification of therapeutic response.

### Classification of therapeutic response

Three categories of therapeutic response, namely Early Treatment Failure (ETF), Late Treatment Failure (LTF) and Adequate Clinical and Parasitological Response (ACPR) were used. This is based on the WHO classification system [7]. At all times during the study, the patient welfare took priority and all the procedures were done in line with the standard guidelines of good practice,

The statistical procedure adapted for the interpretation of the results allowed testing the hypothesis that the proportion of treatment failures was above a certain level in the study area, and, therefore, a decision to change is deemed necessary.

Collaboration with the major institutions in the country ensured the quality of study results. The study team members came from the National Malaria Control Centre, Tropical Diseases Research Centre, Macha Malaria Research Institute, University Teaching Hospital and Chainama Hills College of Health Sciences and District Health Management teams (DHMTs).

### Ethical considerations

The study was conducted in accordance with the standards of good clinical practice.

Ethical clearance was sought from The Tropical Disease Research Centre Institutional Research Committee (#00002911). The study was also cleared by the Central Board of Health Director General's Office and the participating districts. All participants were enrolled after a written consent was obtained from the caretaker. The participants also received incentives to help them during the long follow up days. All information obtained in the study was treated with the confidentiality it deserved.

## Results

Out of the 111 patients enrolled in the two sites, 91 were successfully followed up. Baseline characteristics of participants are outlined in Table 1. The ACPR after 28 days follow up was 100% (95% confidence interval 96.0% to 100%). There were no early treatment failures reported and all the analysable LCF and LPF were due to re-infections. The gametocytes that were reported to be present on Day 0 (368 per microlitre of blood) and Day 2 (336 per microlitre of blood) reduced significantly by Day 7 (80/μl of blood), with none being recorded on Day 21 and Day 28. No clinically detectable drug reactions were reported during the study period. The rate of re-infection was found to be 37% (Chongwe) and 20.8% (Chipata), respectively (Table 2).

**Table 1:** Baseline characteristics of participants

	Chongwe	Chipata
Number Screened	326	627
Parasite Rates (%)*	53.7	30.8
Number Enrolled	54	57
Mean age (months)	22.3	23.5
Female (%)	49.08	54.6
Mean weight	13.7	10.9
Mean Temp, Day 0	37.7	38.7

**Table 2:** Therapeutic response to Co-artesiane^® ^

Variable	Chongwe N = 54		Chipata N = 57	
	
	n	%	n	%
Exclusion	0	0	1	1.8
Loss to follow-up	0	0	6	10.5
Withdrawal	0	0	2	3.5
ETF^U ^	0	0	0	0
LCF^U ^	8	14.8	8	14
LPF^U ^	19	35.2	6	10.5
ACPR^U ^	27	50	39	68.4
Re-infections	20	37	10	17.5
LTF^C ^	0	0	0	0
Inadequate sample	7		3	
Evaluable	47		44	
ACPR^C ^	47	100	44	100

## Discussion

Coartesiane^® ^was found to have excellent 28-day therapeutic efficacy among children weighing less than 10 Kg. This drug has the potential of providing a viable option for children who are not eligible to take Coartem^® ^. There is need, therefore, to continue to evaluate the long-term safety profile of this drug in the younger children. The drug had 28-day ACPR rates superior to those of SP (100% vs 76.1%) being recorded in Zambia in children with similar age and weight characteristics.

The study is limited in nature because it was not a randomized clinical trial which would have been ideal. However, an open label was opted for because there is sufficient data on the efficacy of the tablet formulation of artemether-lumefantrine[8-11].

Further studies are needed to estimate the long-term safety and pharmacological action of this pediatric formulation. This will provide more information for countries where the use of this drug may be contemplated. The gametocyte clearance characteristics were similar to those, which have been recorded with the tablet formulation of artemether-lumefantrine [8,9]. The use of MSP genotyping to differentiate re-infections from recrudescent strains has been useful in evaluating the therapeutic efficacy of the drug.

## Conclusion

Coartesiane^® ^was very effective in treating uncomplicated *P. falciparum *malaria in children weighing less than 10 Kg in Zambia.

## Competing interests

The author(s) declare that they have no competing interests.

## Authors' contributions

PC was responsible for the proposal development, study design, training of research assistants, data analysis and developing of the manuscript. MH was involved in sampling, training of research assistants, field supervision and revision of the manuscript. MK participated in the writing of the manuscript and case management. NS was involved in supervision and manuscript development.
